# Age and sex effects of a validated LC-MS/MS method for the simultaneous quantification of testosterone, allopregnanolone, and its isomers in human serum

**DOI:** 10.1038/s41598-024-78807-3

**Published:** 2024-11-13

**Authors:** Khalisa Amir Hamzah, Leisa-Maree Toms, Nathaniel Kucharski, Julia Orr, Peter Hobson, David S. Nichols, Luke J. Ney

**Affiliations:** 1 School of Psychology and Counselling, Faculty of Health, Queensland University of Technology, 149 Victoria Park Road, Kelvin Grove, Brisbane, 4059 Australia; 2 School of Public Health and Social Work, Faculty of Health, Queensland University of Technology, Kelvin Grove, Australia; 3 The University of Queensland, Queensland Alliance for Environmental Health Sciences, Woolloongabba, QLD Australia; 4 Sullivan and Nicolaides Pathology, 24 Hurworth Street, Bowen Hills, QLD 4006 Australia; 5 Central Science Laboratory, University of Tasmania, Tasmania, Australia

**Keywords:** Neurosteroids, Allopregnanolone, Testosterone, Liquid chromatography tandem mass spectrometry, Age and sex effects, Lipids, Endocrinology

## Abstract

Despite the great relevance of the neurosteroid allopregnanolone and related isomers to various health conditions, quantification typically involves immunoassay, which suffers from serious issues with cross-reactivity of closely related molecules. This article describes the development and partial validation of a liquid chromatography coupled with tandem mass spectrometry assay for the simultaneous quantification of allopregnanolone, pregnanolone, isopregnanolone, epi-allopregnanolone, and testosterone in the human serum of healthy males and females aged 5–85 years. 1-amino-4-methylpiperazine (AMP) was used as a derivatisation reagent to enhance the ionisation signal. Linearity was calculated at 0.99 with a lower limit of quantification of 10.08 pg/mL for allopregnanolone, along with a linearity of 0.98 and a lower limit of quantification of 42.32 pg/mL for testosterone. Application of the method showed sex and age effects across the lifespan for both allopregnanolone and testosterone, whereas a comparative immunoassay for allopregnanolone was not able to detect differences in the same samples. Our partial validation of this method should provide a new tool for researchers to discover the role of allopregnanolone and its isomers in human health, and how it compares to testosterone and sex hormones.

**Supplementary Information:**

The online version contains supplementary material available at 10.1038/s41598-024-78807-3.

Khalisa Amir Hamzah^1*^, Leisa-Maree Toms^2^, Nathaniel Kucharski^2^, Julia Orr^3^, Peter Hobson^3,4^, David S Nichols^5^, Luke J Ney^1^.

^1^School of Psychology and Counselling, Faculty of Health, Queensland University of Technology, Brisbane, Australia.

^2^School of Public Health and Social Work, Faculty of Health, Queensland University of Technology, Kelvin Grove, Australia.

^3^The University of Queensland, Queensland Alliance for Environmental Health Sciences, Woolloongabba, QLD, Australia.

^4^Sullivan and Nicolaides Pathology, 24 Hurworth Street, Bowen Hills 4006, Queensland, Australia.

^5^Central Science Laboratory, University of Tasmania, Australia.

*Corresponding Author: khalisa.amirhamzah@gmail.com, 149 Victoria Park Road, Kelvin Grove, Brisbane Australia 4059.

## Introduction

Allopregnanolone is a stress-related neurosteroid with a primary function in the modulation of gamma-aminobutyric acid (GABA), the chief inhibitory neurotransmitter of the central nervous system^1–4^. It has also been found to be a biomarker in various psychiatric disorders, such as post-traumatic stress disorder (PTSD)^5–7^. Allopregnanolone and its metabolites are closely linked to the regulation of the stress-activated hypothalamic-pituitary-adrenal (HPA) axis, with increases in allopregnanolone levels during acute stress normalising the HPA axis^8^ and decreases in allopregnanolone levels during chronic stress producing behavioural changes^9^. More generally, allopregnanolone has a widespread effect on human immune and health functions^2,10,11^. Despite these important roles in human health, there are limited published assays that accurately and reliably quantify allopregnanolone in samples collected from healthy volunteers.

The first method published to quantify allopregnanolone was determined by radioimmunoassay (RIA). Assay sensitivity was reported at 15–20 pg/tube, and mean allopregnanolone serum levels were reported to fluctuate between 0.79 and 3.69 nmol/L^12^, converting to approximately 248–1,160 pg/mL^13^. Though, RIAs are known to inflate measurements in biomatrices such as blood due to cross-reactivity of closely related metabolites^14^, leading to insufficient discrimination of isomers using immunoassay methods. This issue leads to lack of accuracy in measurement, with similar sex steroid hormones showing lack of identification from precursors and metabolites and even inability to separate menstrual cycle phase^15,16^.

In more recent years, liquid chromatography-tandem mass spectrometry (LC-MS/MS) has been used to more accurately measure allopregnanolone. Mayne, De Bloois^17^ quantified allopregnanolone, pregnanolone, epi-allopregnanolone, pregnenolone, cortisol, and cortisone using a Sciex API5000 triple quadrupole LC-MS/MS system equipped with an EC-C18 analytical column. Serum samples were extracted using ethyl acetate: cyclohexane (1:1, *v: v*). Water with 0.1% formic acid was used as mobile phase A, while methanol with 0.1% formic acid was used as mobile phase B. A lower limit of quantification (LLOQ) was reported as 0.78 ng/mL for allopregnanolone, of which the researchers reported some samples having dropped below and being unable to accurately quantify. This relatively high LLOQ is most likely due to the poor ionisation of allopregnanolone, which can be improved using a derivatisation step^18^.

**Fig. 1:**
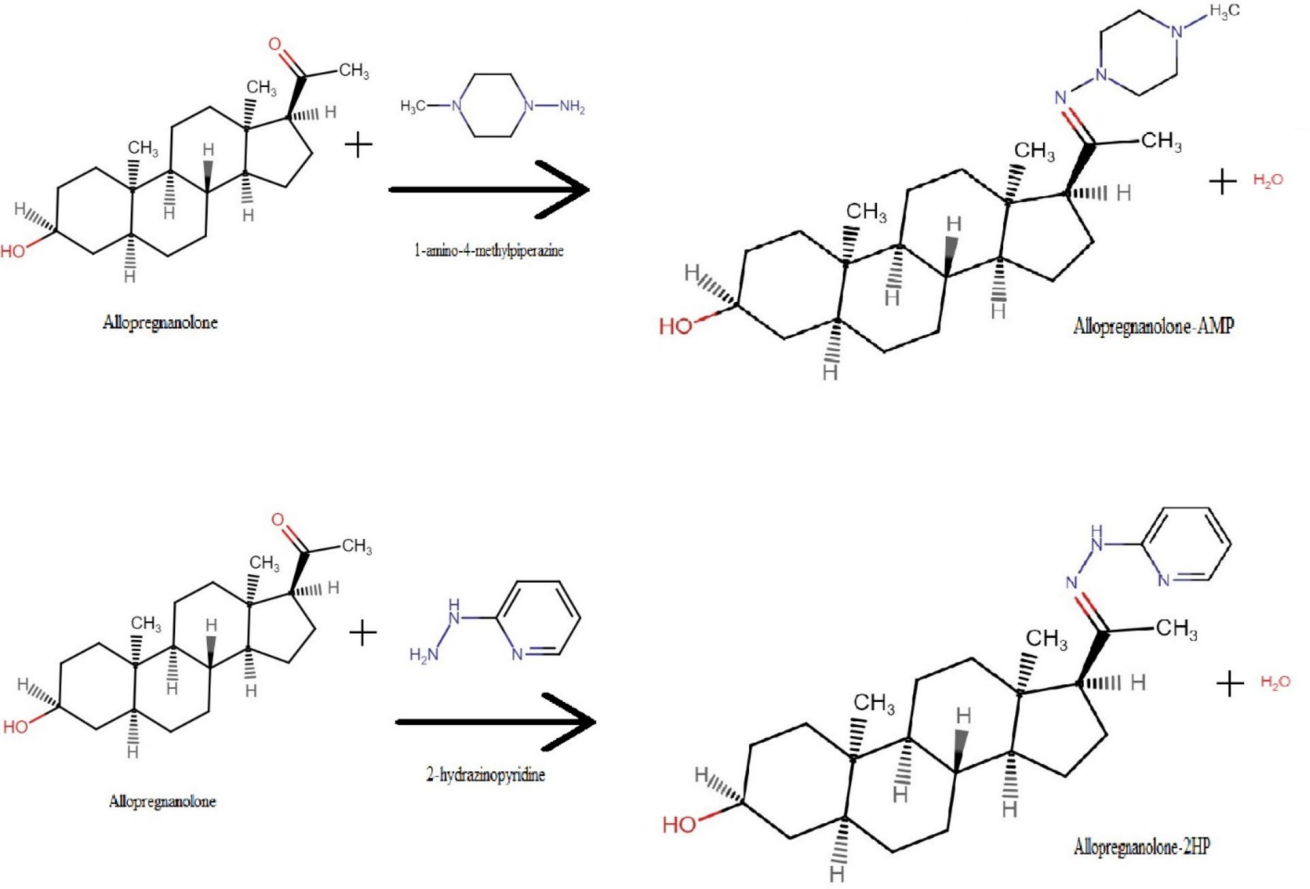
Ketone derivatisation of allopregnanolone by 1-amino-4-methylpiperazine and 2-hydrazinopyridine. AMP = 1-amino-4-methylpiperazine; 2HP = 2-hydrazinopyridine.

Both Lionetto, De Andrés^19^ and Ke, Gonthier^20^ included derivatisation in the development of their assays for a more sensitive quantification of allopregnanolone. Allopregnanolone is a low-abundance analyte, and as such, is often derivatised for quantification to enhance the mass spectrometry ionisation efficiency and improve the chromatographic separation and intensity. The ketone groups of testosterone, allopregnanolone and its isomers can be derivatised with a ketone derivatisation reagent such as 1-amino-4-methylpiperazine (AMP) or 2-hydrazinopyridine (2-HP)^21^ (Fig. 1). The ketone group reacts with the derivatisation reagent to form a new derivative that is more stable and amenable for analysis. The reaction of the ketone and AMP or 2-HP, as hydrazines, forms a hydrazone. This process allows the single ketone groups on allopregnanolone, pregnanolone, and testosterone to neatly derivatise and allow for significantly improved sensitivity.

**Fig. 2:**
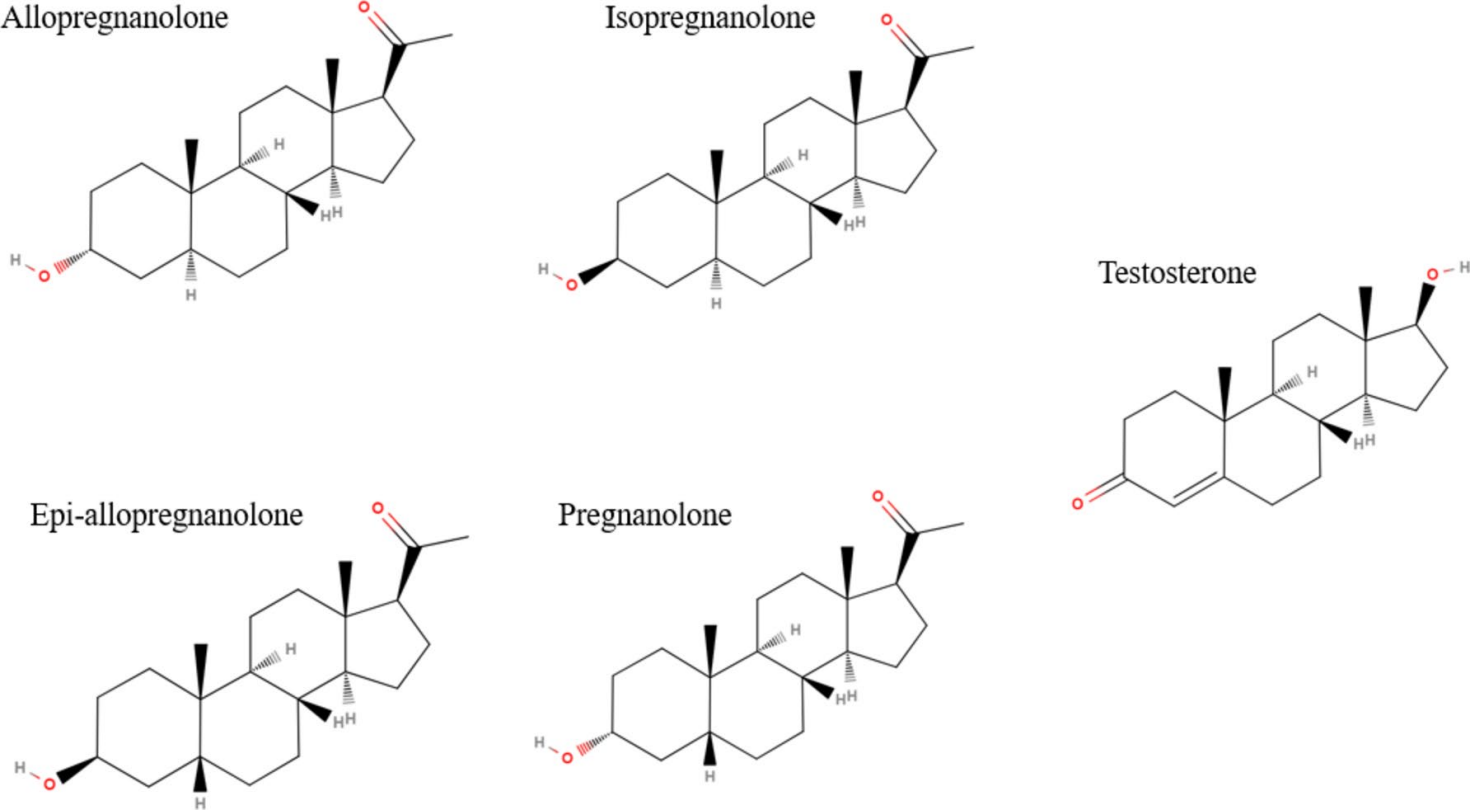
Chemical structures of allopregnanolone, isopregnanolone, epi-allopregnanolone, pregnanolone, and testosterone.

Lionetto, De Andrés^19^ used 2-HP, while Ke, Gonthier^20^ used AMP. Both assays achieved lower LLOQs than previously reported, with Lionetto, De Andrés^19^ achieving 10 pg/mL in plasma and Ke, Gonthier^20^ achieving 5 pg/mL in serum. However, neither method has been independently validated or widely adopted as a routine measurement of allopregnanolone and its isomers. The biosynthesis pathways (Fig. 2) for allopregnanolone and its isomers, along with testosterone, shows that allopregnanolone and its isomers are structurally very similar. Therefore, very sensitive measures are needed for the accurate and reliable quantification of allopregnanolone, controlling for its isomers.

Testosterone was included in the method, given that there is increasing evidence of a sex hormone influence in allopregnanolone and neurosteroid biosynthesis^22,23^, along with activation and modulation of the HPA axis^1^. However, this is the first published method to simultaneously quantify testosterone alongside allopregnanolone and all its isomers. Limited research has expanded to include and investigate testosterone and male sex hormone levels, despite allopregnanolone being a metabolite of a sex hormone, progesterone. Such an assay allows for the exploration of male-abundant sex hormones alongside allopregnanolone concentrations, to expand on the broader relationship between allopregnanolone and sex hormones. Testosterone’s inclusion was also proof of principle that this derivatization assay could be expanded to other related steroid hormones that contain a ketone functional group. Therefore, our group sought to develop a LC-MS/MS assay for the quantification of allopregnanolone, epi-allopregnanolone, pregnanolone, isopregnanolone, and testosterone in human serum.

## Materials and methods

### Chemicals and disposables

The standards allopregnanolone, allopregnanolone-d5, pregnanolone, epi-allopregnanolone, isopregnanolone, testosterone, and testosterone-d3 were purchased from Sapphire Bioscience, Sigma Aldrich, and Novachem. The derivatising reagent 1-amino-4-methylpiperazine (AMP) as well as acetic acid were purchased from Sigma-Aldritch (Missouri, United States). Acetonitrile, isopropanol (propan-2-ol), methanol (MeOH), cyclohexane, ethyl acetate, and distilled water were all LC-grade and were purchased from Thermo Fisher Scientific. Small volume inserts, 2mL polypropylene centrifuge tubes, and HPLC vials (glass) were obtained from Agilent Technologies, Sarstedt Australia, and Thermo Fisher Scientific.

### Standard solutions and calibrators

The chemical purity of the supplied compounds was ≥ 95% for allopregnanolone, allopregnanolone-d5, pregnanolone, epi-allopregnanolone, and isopregnanolone, 98% for testosterone, and ≥ 99% for testosterone-d3. All standards were dissolved in methanol.

The stable isotope labelled internal standards described above were used to quantitate allopregnanolone, pregnanolone, epi-allopregnanolone, isopregnanolone and testosterone via stable isotope dilution. Allopregnanolone-d5 was used as a surrogate labelled standard for allopregnanolone, pregnanolone, epi-allopregnanolone, and isopregnanolone, and testosterone-d3 was used as a surrogate standard for testosterone. Likewise, allopregnanolone-d5 was used in validation measures for allopregnanolone and its isomers, as they share the same transitions on ultra-performance-LC/MS/MS (Supplementary Table 1). Stock standard solutions were mixed to produce a surrogate standard solution (allopregnanolone-d5 5 µg/mL, testosterone-d3 5 µg/mL). This intermediate standard solution was then used to produce a working standard solution (250 ng/mL for each standard) in methanol. 20µL of this standard (i.e., 5 ng of each standard) was added to samples prior to overnight sample extraction for quantitation, or as described below for the determination of method validation parameters.

### Sample preparation

#### Trialled sample preparation methods

Various forms of solid-phase extraction (SPE) and liquid-liquid extraction treatments were compared for maximal optimisation of the sample preparation. SPE treatment was executed after or before derivatisation, eluting with either methanol, acetonitrile, or a solution of 5% (v/v) acetonitrile, 0.5% (v/v) acetic acid, and 94.5% (v/v) water. Both chloroform and hexane liquid-liquid partitioning were also tested as procedures to improve sample extract matrix response. Due to allopregnanolone’s slightly polar properties, the analytes were found in the bottom organic layer with chloroform partitioning (K = 34.90), while they were found in the bottom aqueous layer with hexane partitioning (K = 0.02).

#### Optimised sample preparation method

On the day of the analysis, sera samples were defrosted and analysed with both Enzyme-linked immunosorbent assays (ELISA) and the LC-MS/MS developed method for comparison purposes. Enzyme-linked immunosorbent assay kits for allopregnanolone (ABclonal, General Allopregnanolone ELISA Kit, RK00622) were performed according to manufacturer instructions. Sensitivity is reported to be 9.5 pg/mL, along with a standard curve range of 31.2 pg/mL to 2000 pg/mL.

In preparation for LC-MS/MS analysis, samples were homogenised by vortex and 200 µL was pipetted into multiple 2 mL Eppendorf tubes. Exactly 1.2 mL of 50:50 ethyl acetate: cyclohexane was added as the extraction solvent to each sample, as optimised from previous studies^24,25^. A working solution containing 250 ng/mL of labelled standards (20 µL; 5 ng total) were then added. Each sample was vortexed for one minute and centrifuged at 4 °C at 14,500 rpm for five minutes. After this, the organic layer was transferred to a new tube and an additional 500 µL extraction solvent was added. Once again, these samples were vortexed, centrifuged, and the organic later was transferred to the new tube. Samples were evaporated to dryness using a Martin Christ Speedivac, then derivatised following a previous method^20^. Derivatisation included adding 400 µL 2 mg/mL AMP and 400 µL 1% acetic acid to the dried sample, then heating the sample for 45 min at 37 °C on a heating block.

Then, samples were evaporated to dryness and partitioned using hexane. Briefly, 750 µL 1:2 hexane: methanol was added to the dried samples, then vortexed vigorously. A further 750 µL 100% hexane was added and vortexed, then 250 µL 100% LC-grade water was added and vortexed. The samples were centrifuged 4 °C at 14,500 rpm for five minutes, then the bottom inorganic layer is pipetted into new tubes. Samples were evaporated to dryness again and reconstituted in 100 µL 100% acetonitrile and transferred into small volume inserts. The small volume inserts in ultra-performance liquid chromatography (UPLC) vials were evaporated to dryness and reconstituted in 30 µL 50% acetonitrile as the final solution.

### UPLC separation

An Acquity BEH C18 column (2.1 × 100 mm ×1.7 μm) (Waters Corporation) was utilised with a Nexera X2 UPLC comprising of two binary pumps and a column oven. The mobile phases were 0.1% formic acid in water (Solvent A) and 100% methanol (Solvent B). The pump B concentration was programmed as reported in Lionetto, De Andrés^19^. Briefly, pump B concentration was increased to 60% in the first minute, then increased to 80% by eight minutes. Then, it was maintained for two minutes. It was rapidly increased to 100% within ten seconds, then maintained there for 1 min. It was rapidly decreased to 60% within the next ten seconds, then maintained there for four minutes. The total run-time was 15 min. The flow rate was 0.3 mL/min and oven temperature was 45 °C. Optimal injection volume was 15 µL. Typical retention times for the native analytes are shown in Supplementary Table 1.

### Mass spectrometry detection

A SCIEX QTRAP 6500 triple quadropole MS/MS was used. MS/MS analysis was conducted in multiple reaction monitoring (MRM) mode where positive electrospray ionisation was used. Curtain gas was set to 35, collision gas to 12, IonSpray voltage to 5000, temperature to 550, ion source gas 1 to 55, and ion source gas 2 to 65. Transitions and parameters are given in Supplementary Table 1. Concentration of isopregnanolone, epi-allopregnanolone, allopregnanolone and pregnanolone were calculated based on the measured response of allopregnanolone-d5 surrogate standard. Concentration of testosterone was calculated based on the measured response of the testosterone-d3 surrogate standard.

### Method development

The sample preparation and mass spectrometry parameters were optimised through a series of experiments. Different heating conditions for AMP derivatisation were tested: 60 °C for an hour, 60 °C for 35 min, 37 °C for an hour, and 27 °C for 35 min.

MS/MS method parameters for derivatised testosterone and allopregnanolone were optimised based on those reported from past literature, as seen in Supplementary Table 2. In addition, product ion scans allowed structural information about each derivatised analyte to be obtained, which assisted in optimising the sensitivity for each MRM transition (Supplementary Table 1). Q3 was set to scan from the upper limit of the precursor ion to (m/z) 50.

The resolution of retention times for all allopregnanolone derivatised isomers was confirmed using standard addition to serum samples (Supplementary Table 1) and sample extract injection volumes of 15 µL, 30 µL, and 50 µL were compared.

The solvent for the final reconstitution of samples was assessed; in 100% methanol, 50% methanol, 100% acetonitrile, and 50% acetonitrile were analysed and compared for best sensitivity.

Partial method validation was then completed by spiking human serum samples with the working surrogate standard solution pre-extraction or post-extraction and these samples were compared to solvent only standards, which allowed us to calculate the method limit of detection (LOD), LLOQ, recovery, matrix interference, and percentage of variation (CV; see methods below for details on these procedures).

### Validation

#### Linearity

Triplicate serum samples were spiked with the labelled surrogate standard solution at concentrations of 10 ng/mL, 5 ng/mL, 2.5 ng/mL, 1.25 ng/mL, 640 pg/mL, 320 pg/mL, 160 pg/mL, 80 pg/mL, 40 pg/mL, and 20 pg/mL to establish linearity. A standard curve was calculated, and the resulting R^2^ value is an indication of the method linearity. These levels were used to establish a range from the high-level spike concentration to an estimate of the lower limit of quantification (LLOQ).

#### Intra-assay precision, limit of detection, and limit of quantification

Precision estimates were obtained with seven replicate samples for low, medium, and high concentrations (Low: 10 pg/mL for allopregnanolone-d5, 50 pg/mL for testosterone-d3; Med: 200 pg/mL for allopregnanolone-d5 and testosterone-d3; and High: 10 ng/mL for allopregnanolone-d5 and testosterone-d3, respectively). Precision was calculated as the percentage covariate of variation (CV). The limit of detection (LOD) was calculated based on the data from the Low replicate samples. The LOD was determined as three times (95% confidence interval) the standard deviation of the Low replicate samples. According to C.3.1 in EU regulations (Regulation 333/2007), the LLOQ was defined as two times the LOD level.

#### Matrix interference and recovery

Sample extract matrix suppression, sample recovery and total method bias were estimated by spiking the stable isotope labelled surrogate standards into multiple replicates of solvent (methanol), pre-extraction serum samples and post-extraction serum sample extracts. We further investigated suppression effects of the matrix and total sample preparation procedures by dividing the peak areas of the post-extracted samples by the peak areas from the pure standard mixtures, then multiplying by 100 (matrix effect suppression; MES). Also, the peak areas of the pre-extracted samples were divided by the peak areas from the pure standard mixtures, then multiplied by 100 (total bias; TB). Sample recovery (REC) was estimated as the division of the peak areas from the pre-extracted samples by the peak areas from the post-extracted samples, multiplied by 100^27^.

#### Carry-over effects

No carry-over effects were observed. High concentrations (10 ng/mL) of allopregnanolone, allopregnanolone-d5, testosterone, and testosterone-d3 were spiked into samples, with blank samples assessed immediately after to examine carry-over. For both the native and labelled analytes, no analyte signal in the following blank samples were detected. This is likely due to the long washout period in the LC-MS/MS method, and the extensive sample clean up after derivatisation.

### Sample collection and storage

All samples were collected between 2021 and 2022 from routine blood collections at multiple pathology laboratories in Australia (Sullivan Nicolaides Pathology, Australia). Samples were collected in serum separating tubes and immediately centrifuged and stored at – 20 °C. Sera was thawed for sample pooling and refrozen at – 20 °C, to be thawed again only for sample analysis preparation.

### Participant serum samples

Blood samples were collected using the methods described in Toms, Harden^28^, which involved collaboration with a key national pathology laboratory in Australia (Sullivan Nicolaides Pathology, Australia). These samples include archived pooled and de-identified human blood sera in an ongoing series of biomonitoring studies from 2002 to current^29,30^. This study was approved by the local Human Research Ethics Committee (approval number 1086) and conducted in accordance with the relevant guidelines and regulations. Additionally, informed consent was obtained from all participants and/or their legal guardians.

The samples have been described in Amir Hamzah, Toms^31^. Briefly, samples were stratified by male and female sex in seven age ranges (Table 1), with two pooled clusters of 25 participants in each stratified bracket. Thus, 50 participant samples are in each sex-stratified age range group and 28 variable groups in total (7 age range * 2 sex * 2 pooled clusters). A total of 700 serum samples were collected from Australians aged 5–85 years old, surplus to the requirements of ongoing studies at Sullivan Nicolaides Pathology, Australia. Pooled sera were available in 2 mL in each stratified sample and were aliquoted from a larger sample base at Sullivan Nicolaides Pathology, Brisbane. Due to the de-identified nature of the collection procedure, specific demographics of participants are not provided, such as participant body-mass index, ethnicity, general health, and medicine use.

**Table 1:** Average age (years) of participants across stratifications of sex and age range.

Age Range (years)	Males	Females	Average
5–15	11.94	11.51	11.72
15–30	21.38	23.76	22.57
30–45	37.34	35.96	36.65
45–60	52.71	52.57	52.64
60–75	67.89	67.28	67.58
75–85	79.29	79.91	79.60
85+	88.66	88.29	88.48

Standard deviations are not reported because only average age values for each pool were recorded. Each cell in male and female columns contains serum samples from two pooled clusters of 25 participants, equalling 50 participant samples per cell.

## Results and discussion

### Method development

The peak intensity of non-derivatised and derivatised samples using either AMP or 2-HP were first compared. We found that AMP-derivatised samples were 230-times more sensitive than non-derivatised samples (Supplementary Fig. 1). However, derivatisation by 2-HP was unable to be completed, as it crystallised multiple times during sample preparation and was unable to be analysed. However, successful derivatisation by 2-HP has previously been published^19,32,33^. These methods were preceded by SPE, which was ultimately not included in the current method. It may be suggested that the sample in the current method was not at an optimal concentration or purification prior to the attempt to derivatise by 2-HP, as simple lipid extraction was performed instead of SPE. Derivatisation using only AMP was therefore validated throughout the study.

Different heating conditions for AMP derivatisation were tested as outlined in Sect. Method development, with samples heated at 37 °C for 30 min reporting the best peak intensity and shape, though only for testosterone (Supplementary Fig. 2). The size of the sample extract injection volume was also optimised, where injections of 15 µL showed best peak intensity and shape (Supplementary Fig. 3). Likewise, the final sample extract solvent was tested for best sensitivity, where extracts reconstituted in 30 µL of 50%, 25%, and 75% acetonitrile showing similar peak intensity, compared to methanol (Supplementary Fig. 4). Overall, a final solvent reconstituted in 30 µL of 50% acetonitrile was chosen, as it was the most reliable on column.

However, when method validation was initially undertaken, progressive loss of signal was observed over each batch run of this analysis, most likely due to an observed build-up of residue found in the column (Supplementary Fig. 5). To resolve this issue, we sought to optimise sample preparation by cleaning the sample extracts using solid-phase extraction (SPE) or further liquid extraction. Eluent solvents and SPE before or after derivatisation were tested (Supplementary Fig. 6). Both acetonitrile and acetic acid as eluents resulted in more noise than recognised peaks. Therefore, these eluents were either ineffective in eluting the bound analytes, or the analytes may have failed to initially bind to the SPE column and were subsequently lost in the wash step. Contrastingly, methanol allowed for moderate analyte recovery. SPE before derivatisation resulted in comparatively better analyte signal to noise ratio than samples from SPE after derivatisation.

Though, liquid-liquid partitioning with either chloroform or hexane resolved the batch signal loss issues and resulted in better peak shape. Comparison of results from chloroform and hexane partitioning show that hexane allowed for comparatively more pronounced peak shape and intensity (Supplementary Figs. 7–8). This led to ethyl acetate: cyclohexane extraction and protein precipitation, AMP derivatisation, and hexane liquid-liquid partitioning being used in the final sample preparation process.

#### Chromatographic separation

**Fig. 3:**
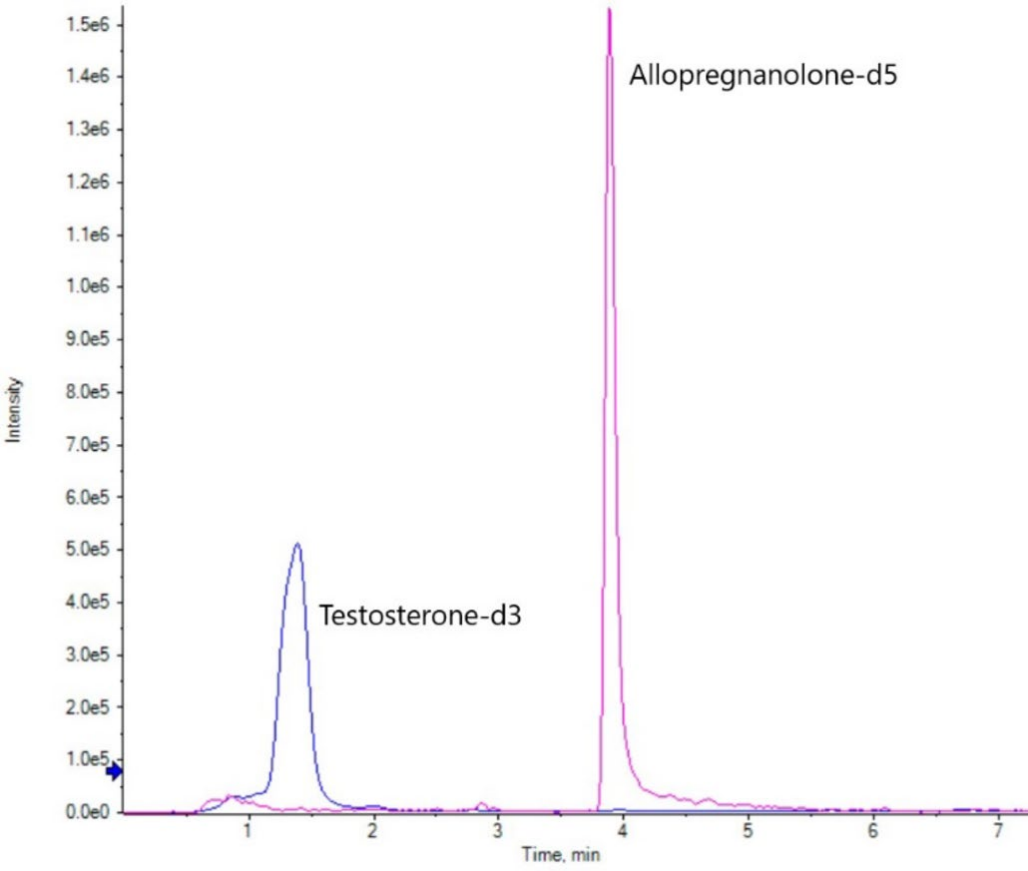
UPLC-MS/MS chromatographic separation of AMP derivatised labelled surrogate standards (Testosterone-d3, blue; Allogregnanolone-d5, pink) from an optimised method serum extract. Concentrations reflect the medium level method validation spike (200 pg/mL in serum).

Figure 3 visualises chromatographic separation of the two derivatised stable isotope labelled standards in a serum sample, allopregnanolone-d5-AMP for allopregnanolone, pregnanolone, isopregnanolone, and epi-allopregnanolone, and testosterone-d5-AMP for testosterone. Spiked levels of the stable isotope labelled surrogate standards in Fig. 3 reflect the medium level method validation spike (200 pg/mL in human serum, 100 pg on-column). It should be noted that there is some significant tailing of allopregnanolone-d5 in the 200 pg/mL spikes. Though this effect was reduced in lower concentration spikes, this limitation indicated how the low abundance of the analyte affected the specificity of the method for heavy-labelled allopregnanolone.

**Table 2:** Linearity and detection limits for AMP derivatised allopregnanolone and testosterone stable isotope labelled standards in serum obtained from the validation data using the optimised method.

Compound	Calibration Curve	*R* ^2^	LOD (pg/mL)	LLOQ (pg/mL)	Retention Time (min)
Allopregnanolone-d5	*y* = 3376.7x^2–12572x + 13,506	0.993	5.04	10.08	3.9
Testosterone-d3	*y* = 1212.3x^2–5825.2x + 6989.1	0.981	21.31	42.32	1.4

LOD = limit of detection, LLOQ = lower limit of quantification.

**Fig. 4:**
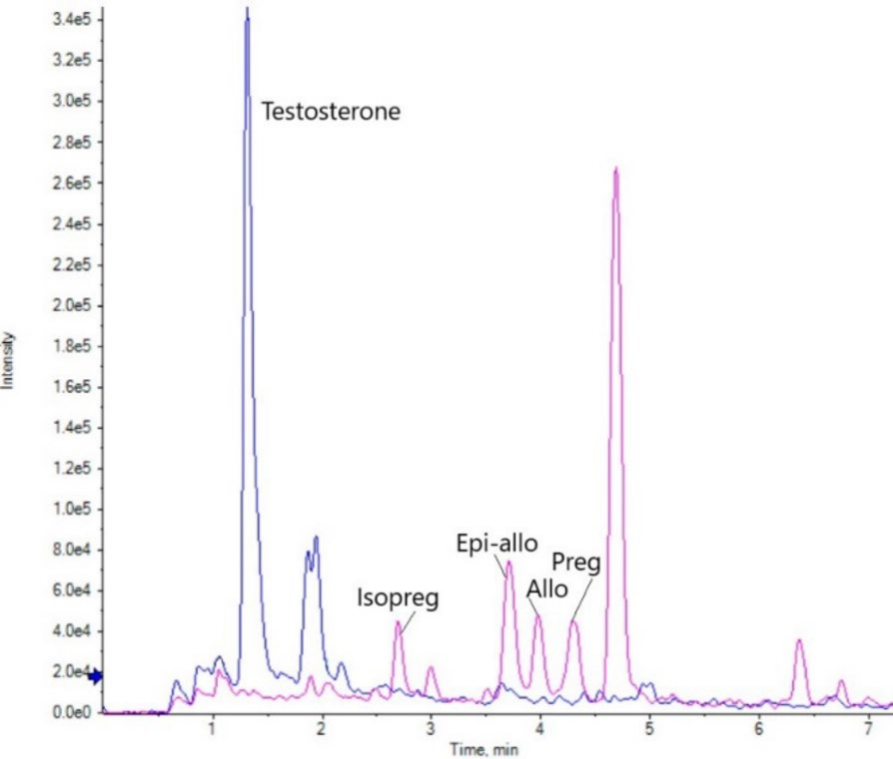
UPLC-MS/MS chromatographic separation of the AMP derivatised native analytes of Allopregnanolone isomers (pink) and Testosterone (blue) from an optimised method serum extract. Isopreg = isopregnanolone, Epi-allo = epi-allopregnanolone, Allo = allopregnanolone, preg = pregnanolone.

In Fig. 4, chromatographic separation of native levels of each analyte are visualised from serum samples. The method is successfully able to separate allopregnanolone from its isomers, with resolution and confirmation of allopregnanolone from the isomers isopregnanolone, epi-allopregnanolone and pregnanolone achieved using standard addition (not visualised). In addition, there was a consistent large signal at 4.7 min within the MRM transition chromatogram (m/z) 416 > 99 from human serum extracts, which was also resolved from the analytes of interest within the scope of this method. The identity of this compound remains under investigation.

#### Linearity, LOD, and LLOQ

A summary of the analyte linearity, LODs, and LLOQs are detailed in Table 2. R^2^ values were above 0.98 for allopregnanolone and testosterone. Mayne et al. (2021) report a linear range from 0.78 to 100 ng/mL for allopregnanolone, without derivatisation. Meanwhile our group report a linear range from 20 pg/mL to 10 ng/mL, which allows for a more holistic view of allopregnanolone concentrations in serum, accounting for the effect of psychiatric and health conditions that may decrease allopregnanolone serum levels substantially. The current study compares similarly to that of Ke et al. (2017), of whom the derivatisation procedure is referenced. Both groups report adequate LLOQs for allopregnanolone, though Ke et al. (2017) did not analyse testosterone. Van Nuland et al. (2019) reported an LLOQ of 10 pg/mL for testosterone and a linear range from 10 pg/mL to 7.5 ng/mL. Additionally, Bui et al. (2013) reported a limit of quantification of 28.8 pg/mL for testosterone. Choi et al. (1999) measured testosterone and epitestosterone through derivatisation of two ionisable positions with the ketone and hydroxyl groups, with derivatisation at the ketone position resulting in the better mass spectrometry results. Testosterone was quantified from 2.03 ng/g to 2.54 ng/g in male hair, and from 0.88 ng/g to 1.66 ng/g in female hair. Furthermore, to our knowledge, there are no published assays to date that simultaneously quantify allopregnanolone, its isomers, and testosterone with a ketone-derivatisation.

#### Matrix interference and recovery

Matrix interference, recovery, and other validation parameters are reported in Table 3. Recovery for allopregnanolone remained high, though recovery for testosterone at low level concentration dropped. The low-level spike concentration at 0.05 ng/mL was very close to the LLOQ for testosterone, and we did not observe any issues at levels higher than the lowest attempted concentration. Reference to other published methods suggests that specific assays for the single quantification of testosterone, such as Van Nuland et al. (2019), are able to achieve lower levels of quantification and higher recovery data at low concentrations. This may be expected as derivatization of testosterone is not required to improve ionisation. CVs for serum analytes were generally below 20%, except for medium-spike testosterone, which was slightly higher. This method performance was adequate as per reported guidelines for bioanalytical method validation for variation in human biomatrices^24,34^.

**Table 3:** Validation parameters, recovery, and matrix interference for low, medium, and high levels of AMP derivatised allopregnanolone and testosterone stable isotope labelled standards in serum.

Compound	Validation Concentration (ng/mL)	CV (%)	Recovery (%)	MES (%)	Total Bias (%)
Allopregnanolone	0.01	16.8	91.9	101.5	93.3
	0.2	18.5	102.2	-	-
	10	14	104.9	78.8	82.5
Testosterone	0.05	14.2	67.7	144.1	97.6
	0.2	21.9	80.3	-	-
	10	13	102.4	116.4	119.2

CV = coefficient of variation, MES = matrix effect suppression. MES values above 100% indicate that signal was enhanced with the biomatrix. Total bias values lower than 100% indicate procedural losses across the whole procedure.

#### Serum samples measured by ELISA

**Fig. 5:**
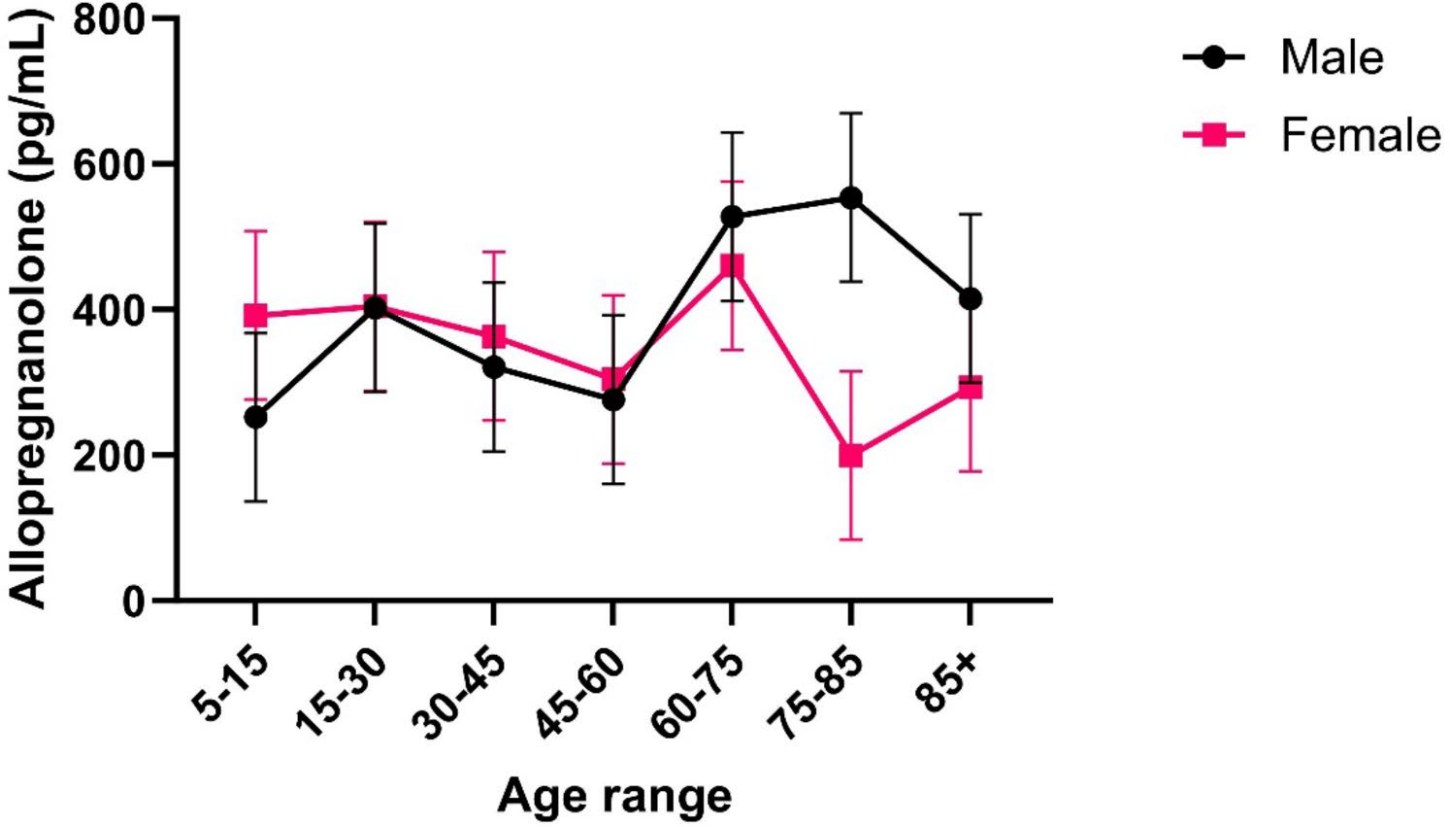
Quantification of allopregnanolone from serum samples by the ELISA method. Error bars represent standard error of the mean.

**Fig. 6:**
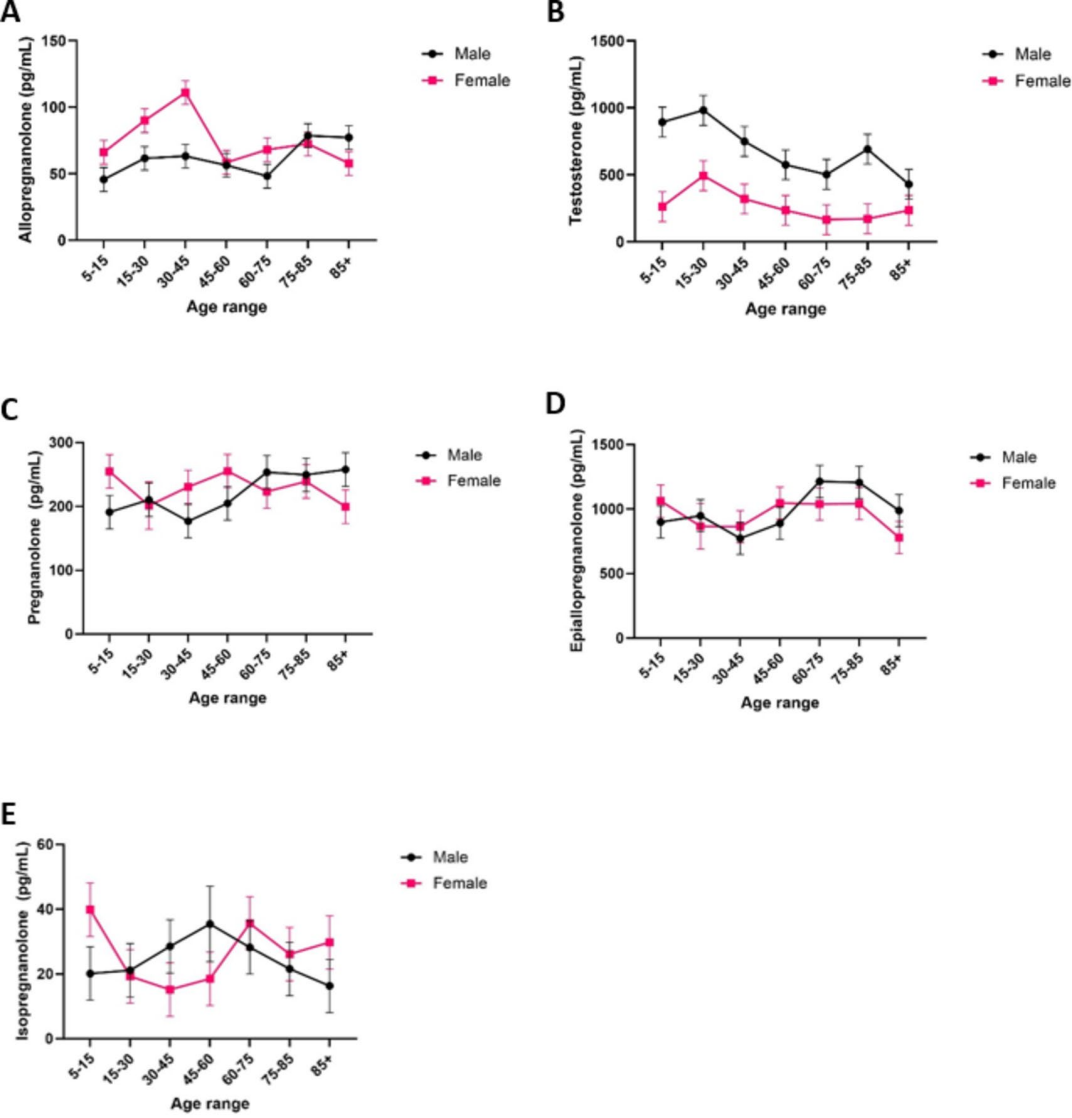
Quantification of allopregnanolone (Panel **A**), testosterone (Panel **B**), pregnanolone (Panel **C**), epi-allopregnanolone (Panel **D**), and isopregnanolone (Panel **E**) from serum samples by UPLC-MS/MS. Error bars represent standard error of the mean.

In general, ELISA methodology greatly over-estimated the quantification of allopregnanolone, in comparison to our mass spectrometry method (Figs. 5 and 6 respectively). Without controlling for age range or sex effects, results for the quantification of allopregnanolone by ELISA ranged from 80.66 pg/mL to 789.34 pg/mL. This suggests a lack of specificity of the ELISA method, most likely due cross-reactivity of allopregnanolone detection by the ELISA method with the isomers of allopregnanolone. This hypothesis is supported by the results from epi-allopregnanolone and pregnanolone by UPLC-MS/MS in the current study, where their abundance was much higher compared to allopregnanolone (Fig. 6, Panels A and C-E). When testing for age range and sex effects, quantification by ELISA suggests that allopregnanolone is more abundant in males than females, which is also seen in quantification by UPLC-MS/MS. Visual inspection of the ELISA results also suggest that allopregnanolone serum levels has a slight upwards trend in females over age, while males have a slight downwards trend over age.

#### Serum samples measured by UPLC-MS/MS

Results from the UPLC-MS/MS method showed the predicted sex and age effects for allopregnanolone (Fig. 6, Panel A), its isomers (Fig. 6, Panels C-E), and testosterone (Fig. 6, Panel B). The total UPLC-MS/MS values for allopregnanolone and its isomers are slightly increased in comparison to the maximum level of allopregnanolone found by ELISA, due to the large epi-allopregnanolone serum levels found by UPLC-MS/MS. Therefore, the over-inflated allopregnanolone levels found by ELISA can be partially explained by cross-reactivity with allopregnanolone isomers, or perhaps cross-reactivity with only certain isomers.

As allopregnanolone is a metabolite of progesterone (a sex hormone, as is testosterone), we would have expected hormonal changes across the lifespan, such as puberty in early life, early adulthood at peak fertility, perimenopause, and menopause. Accordingly, we found that both allopregnanolone and testosterone peaked in late adolescence and/or early adulthood for females and males, respectively. In contrast, pregnanolone, epi-allopregnanolone and isopregnanolone did not show these effects, supporting the hypothesis that cross reactivity of the ELISA assay with these higher abundant isomers reduced the ability for that method to discriminate allopregnanolone levels across sex and age.

## Conclusion

In summary, we developed a UPLC-MS/MS method for simultaneously quantifying allopregnanolone, testosterone, epi-allopregnanolone, isopregnanolone, and pregnanolone in serum. We were able to separate allopregnanolone from its isomers using this method. Extensive optimisation of the method was executed, including derivatisation of the analytes to increase ionisation efficiency, sample preparation optimisation including SPE and liquid-liquid partitioning and parameter optimisation (heating, injection volume, and final solvent reconstitution). It was found that AMP derivatisation with hexane liquid-liquid partitioning, with original heating of the derivatisation reagent at 37 °C for 45 min, injected at 15 µL in a final solvent of 50% acetonitrile showed the most efficient optimisation of the method. Our method is linear with low picogram detection limits for allopregnanolone, though slightly higher but still appropriate detection limits for testosterone. Given the higher abundance of epi-allopregnanolone and pregnanolone, our data suggests that the lack of age and sex effects of the allopregnanolone ELISA data, when compared to our UPLC-MS/MS assay, may be due to cross-reactivity within the ELISA method. However, further comparisons between ELISA and UPLC-MS/MS methods for epi-allopregnanolone and pregnanolone are required to test this directly. Allopregnanolone seems to have age and sex effects across the lifespan, though further research is encouraged to explicitly investigate this.

## Electronic supplementary material

Below is the link to the electronic supplementary material.

Supplementary Material 1.

## Data Availability

The data that support the findings of this study are available in the supplementary material. However, restrictions apply for data according to the age and sex effects available from Sullivan Nicolaides Pathology, Australia. These data were used under licence for the current study and so are not publicly available. Upon reasonable request, additional data may be available through correspondence with the authors (please contact luke.ney@qut.edu.au for data requests), with permission from Sullivan Nicolaides Pathology, Australia. Otherwise, most data generated and analysed during this study are included in this published article.
