# Supplementary material for: Age and sex effects of a validated LC-MS/MS method for the simultaneous quantification of testosterone, allopregnanolone, and its isomers in human serum
Source: Sci Rep. 2024 Nov 13;14:27777. doi: 10.1038/s41598-024-78807-3 (PMC11561161; doi:10.1038/s41598-024-78807-3)
Supplement: Supplementary file 1 — Supplementary Material 1. [file 41598_2024_78807_MOESM1_ESM.docx]

**Supplementary Figures**

**Supplementary Figure 1**

*Comparison of UPLC-MS/MS chromatograms from AMP-derivatised (Blue) and non-derivatised (red) serum sample extracts identifying Allopregnanolone (Allo) and Testosterone (Testo) analytes.*


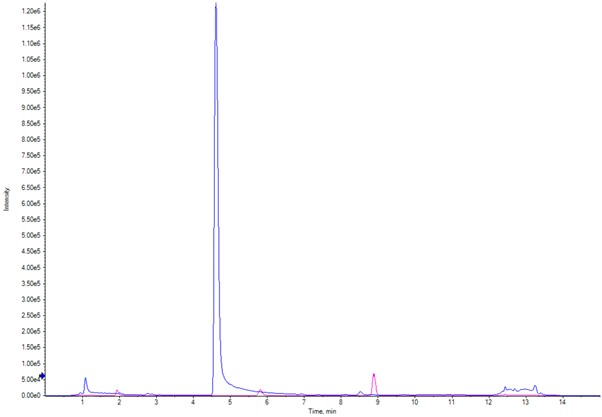


Testo-ND

Testo-AMP

Allo-ND

Allo-AMP

*Note.* AMP = 1-amino-4-methylpiperazine, ND = non-derivatised.

**Supplementary Figure 2**

*Effect of derivatisation reaction heating conditions for AMP reagent derivatisation of Testosterone*


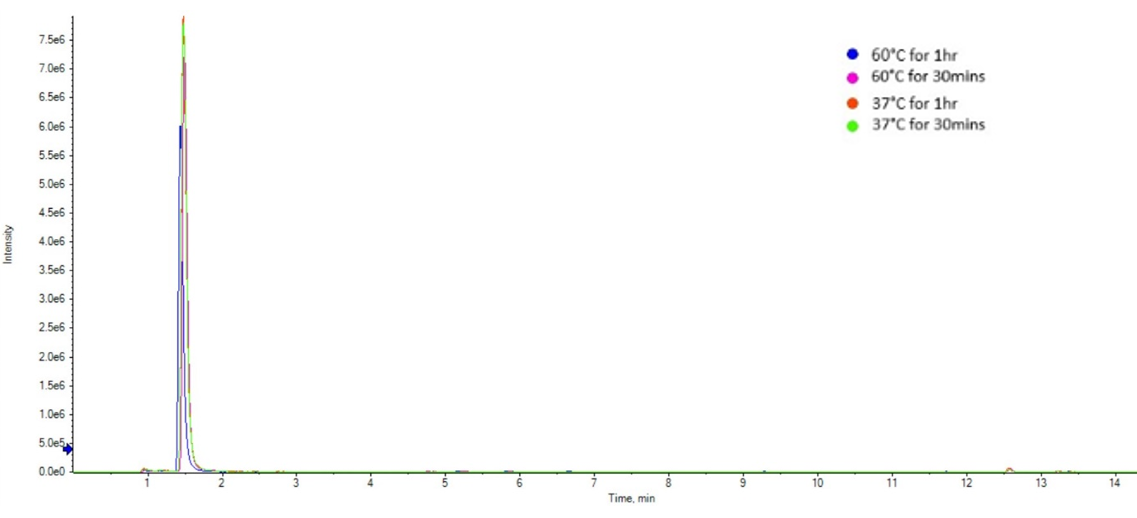


**Supplementary Figure 3**

*Injection volume optimisation*


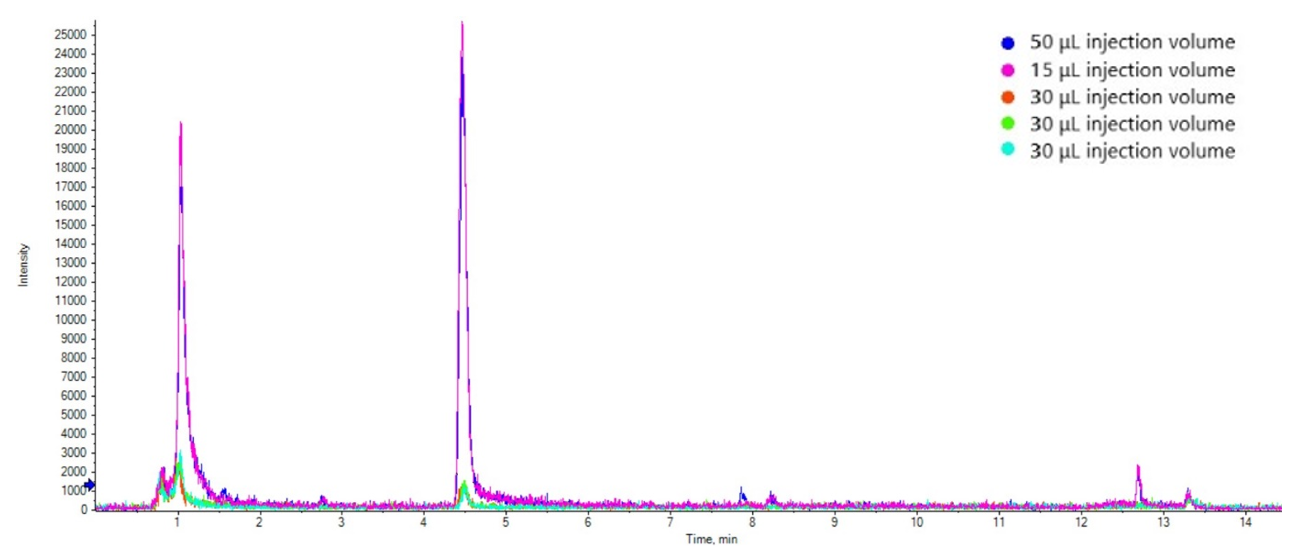


**Supplementary Figure 4**

*Final solvent optimisation*


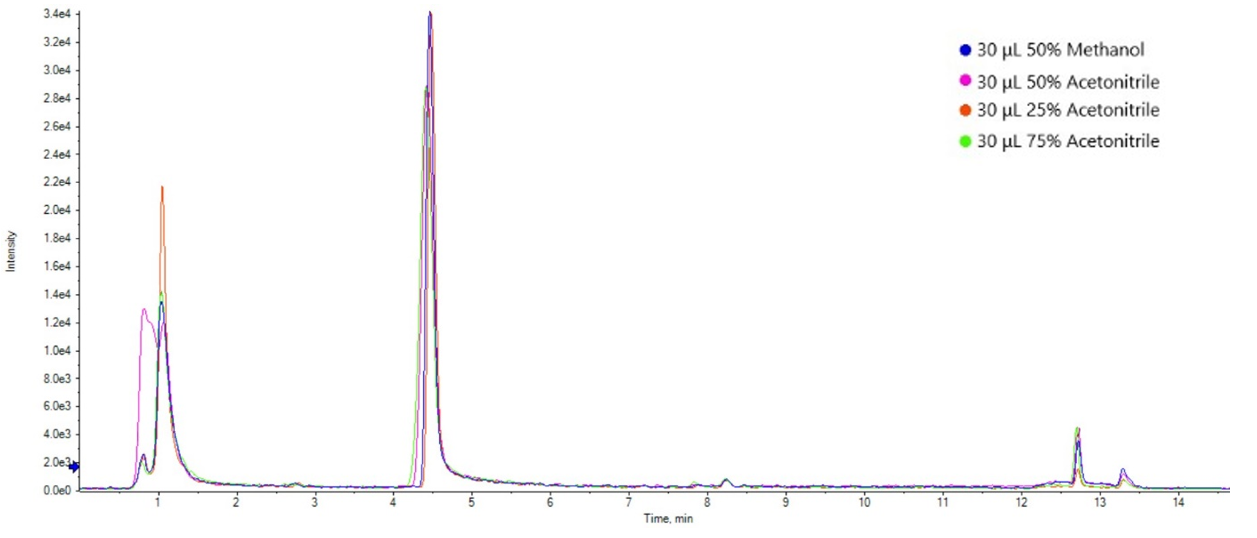


**Supplementary Figure 5**

*Signal loss over batch runs*


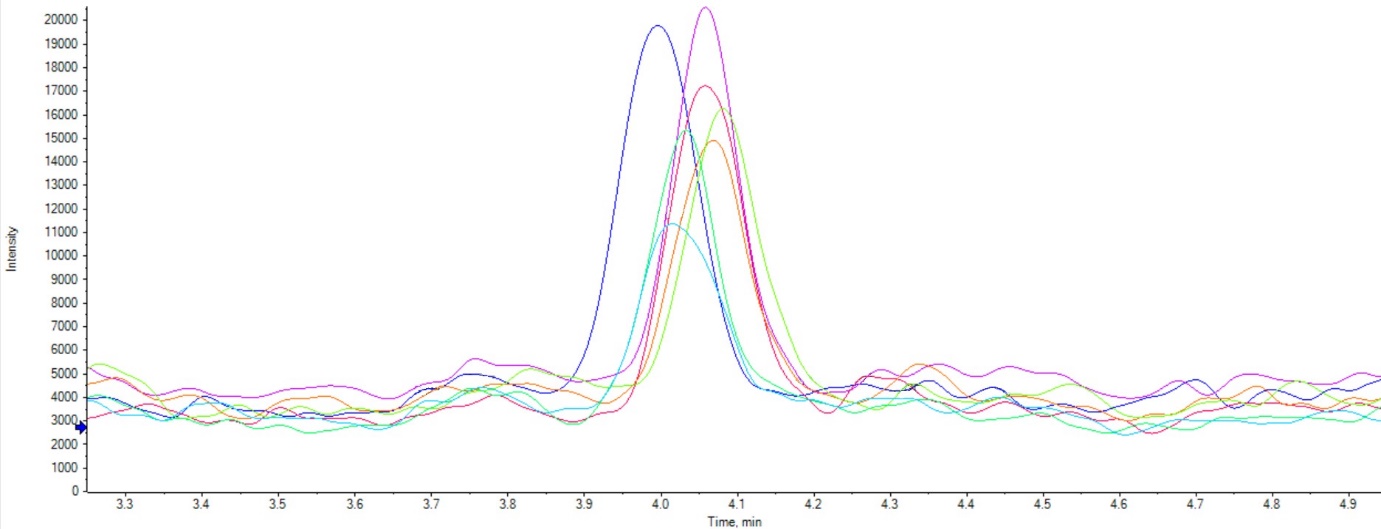


*Note.* Separate runs indicated by colours. All runs received the same method.

**Supplementary Figure 6**

*Solid phase extraction (SPE) conditions*


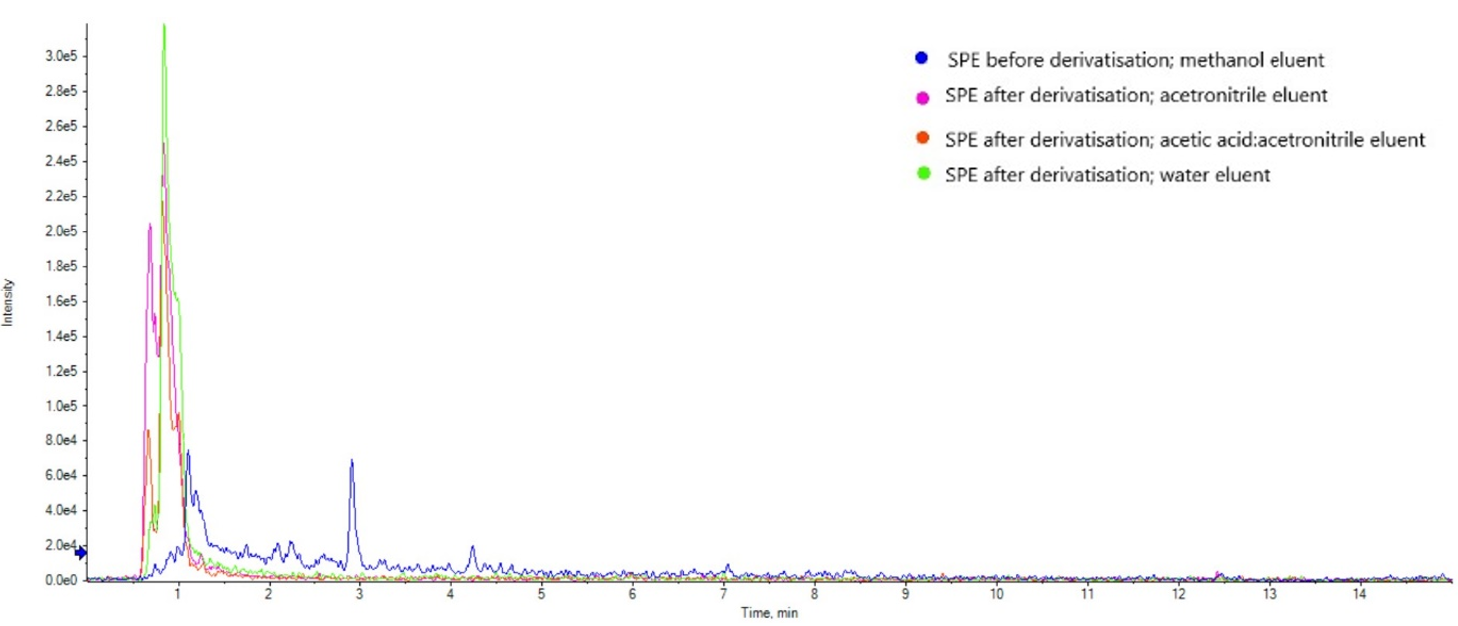


**Supplementary Figure 7**

*Chloroform partitioning results*


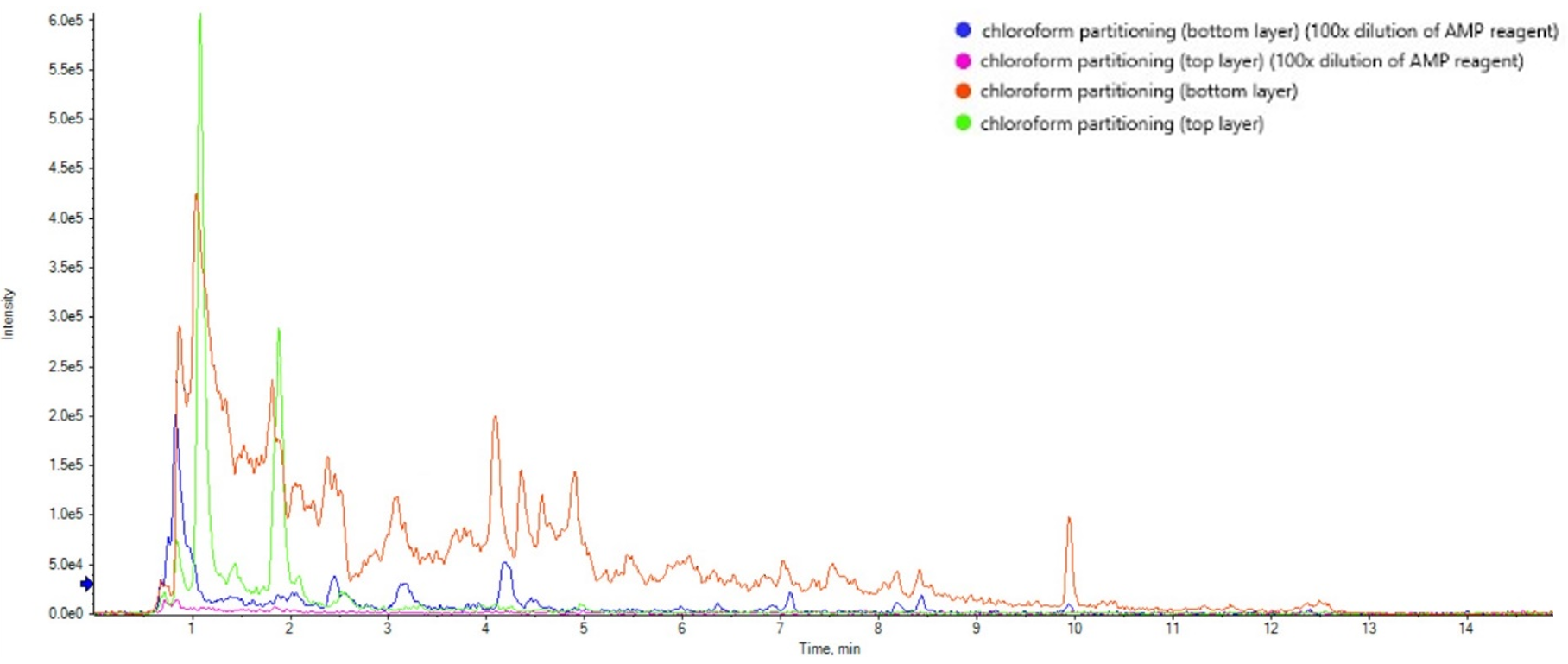


**Supplementary Figure 8**

*Hexane partitioning results*


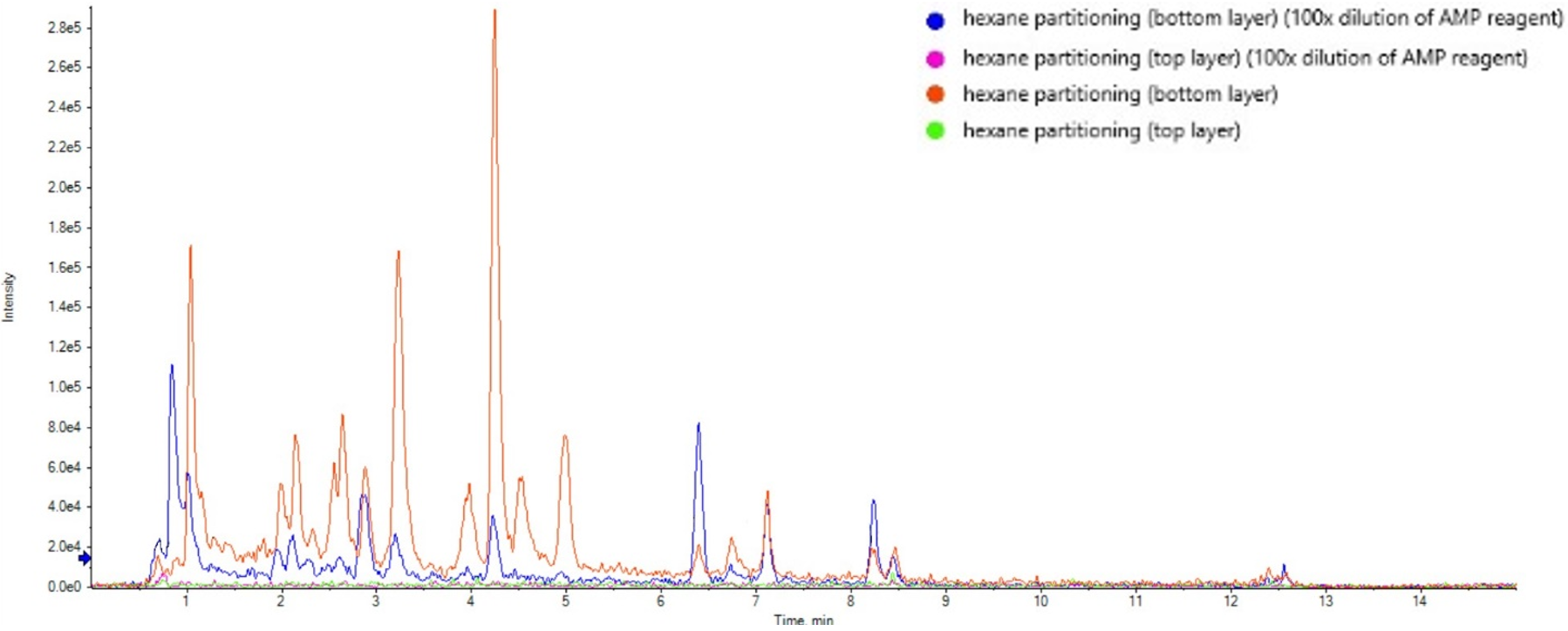


**Supplementary Tables**

**Supplementary Table 1**

*Characteristic retention times together with multiple reaction monitoring (MRM) transitions and parameters for AMP-derivatised analytes investigated from serum samples.*

| Analyte | Retention Time  (min) | MRM Transition  Precursor / Product Ion (m/z) | | | DP (volts) | EP (volts) | CE (volts) | CXP (volts) |
| --- | --- | --- | --- | --- | --- | --- | --- | --- |
| Allopregnanolone | 3.9 | *416* | *🡪* | *99* | 57 | 6 | 35 | 4.9 |
| Epi-Allopregnanolone | 3.7 | *416* | *🡪* | *99* | 57 | 6 | 35 | 4.9 |
| Isopregnanolone | 2.7 | *416* | *🡪* | *99* | 57 | 6 | 35 | 4.9 |
| Allopregnanolone-d5 | 3.9 | *421* | *🡪* | *99* | 57 | 6 | 35 | 4.9 |
|  |  |  | *🡪* | *70* | 57 | 6 | 35 | 4.9 |
|  |  |  | *🡪* | *58* | 57 | 6 | 35 | 4.9 |
|  |  |  | *🡪* | *56* | 57 | 6 | 35 | 4.9 |
| Pregnanolone | 4.3 | *416* | *🡪* | *99* | 70 | 6.3 | 45 | 3.1 |
| Testosterone | 1.3 | *386* | *🡪* | *99* | 57 | 6 | 35 | 3 |

DP = declustering potential, EP = entrance potential, CE = collision energy, CXP = collision cell exit potential.

**Supplementary Table 2**

*Optimisation of multiple reaction monitoring (MRM) method parameters for AMP derivatised Allopregnanolone and Testosterone analytes.*

| **Method code** | **DP** | **EP** | **CE** | **CXP** |
| --- | --- | --- | --- | --- |
| Testo_original | 67 | 6 | 42 | 4 |
| Testo_parameters_1 | 61 | 5.7 | 49 | 4.9 |
| Testo_parameters_2 | 57 | 6.3 | 39 | 3.1 |
| Testo_parameters_3 | 72 | 6.3 | 45 | 3.1 |
| Testo_parameters_4 | 69 | 6 | 42 | 4 |
| Testo_parameters_5 | 64 | 6 | 35 | 4.9 |
| Allo_original | 67 | 6 | 42 | 4 |
| Allo_parameters_1 | 57 | 6 | 35 | 4.9 |
| Allo_parameters_2 | 61 | 5.7 | 49 | 4.9 |
| Allo_parameters_3 | 64 | 6.3 | 39 | 3.1 |
| Allo_parameters_4 | 72 | 6.3 | 45 | 3.1 |
| Allo_parameters_5 | 69 | 6 | 42 | 4 |

*Note.* Method codes were used to differentiate between multiple parameter conditions. "Testo_" refers to Testosterone. "Allo_" refers to Allopregnanolone. “_original” refers to the parameters taken from existing literature (16-19). DP = declustering potential, EP = entrance potential, CE = collision energy, CXP = collision cell exit potential.
